# Metabolic Syndrome and Subclinical Hypothyroidism: A Type 2 Diabetes-Dependent Association

**DOI:** 10.1155/2018/8251076

**Published:** 2018-07-29

**Authors:** Valmore Bermúdez, Juan Salazar, Roberto Añez, Milagros Rojas, Viviana Estrella, María Ordoñez, Maricarmen Chacín, Juan Diego Hernández, Víctor Arias, Mayela Cabrera, Clímaco Cano-Ponce, Joselyn Rojas

**Affiliations:** ^1^Universidad Simón Bolívar, Facultad de Ciencias de la Salud, Barranquilla, Colombia; ^2^Endocrine and Metabolic Diseases Research Center, School of Medicine, University of Zulia, Maracaibo, Venezuela; ^3^Universidad de Especialidades Espíritu Santo, Cantón de Samborondón, Provincia de Guayas, Ecuador; ^4^Grupo de Investigación Altos Estudios de Frontera (ALEF), Universidad Simón Bolívar, Colombia; ^5^Lone Star College, Biology Department, Tomball, TX 77381, USA; ^6^Division of Pulmonary and Critical Care Medicine, Brigham and Women's Hospital and Harvard Medical School, Boston, MA 02115, USA

## Abstract

**Introduction:**

Subclinical hypothyroidism (ScH) is an endocrine alteration that is related to cardiovascular risk factors, including those categorized as components of the Metabolic Syndrome (MS). However, findings in prior reports regarding an association between these alterations are inconsistent. The purpose of this study was to determine the relationship between both entities in adult subjects from Maracaibo City, Venezuela.

**Materials and Methods:**

The Maracaibo City Metabolic Syndrome Prevalence Study is a descriptive, cross-sectional study with random and multistage sampling. In this substudy, 391 individuals of both genders were selected and TSH, free T3, and free T4 tests were performed as well as a complete lipid profile, fasting glycaemia, and insulin blood values. ScH was defined according to the National Health and Nutrition Examination Survey (NHANES) criteria: high TSH (≥4.12mUI/L) and normal free T4 (0.9-1,9 ng/dL) in subjects without personal history of thyroid disease. MS components were defined according to IDF/AHA/NHLBI/WHF/IAS/IASO-2009 criteria. A multiple logistic regression analysis was used to assess the relationship between MS components and ScH diagnosis.

**Results:**

Of the evaluated population, 10.5% (n=41) was diagnosed with ScH, with a higher prevalence in women (female: 13.6% versus male: 7.7%; *χ*2=3.56, p=0.05). Likewise, 56.1% (n=23) of the subjects with ScH were diagnosed with MS (*χ*2=4.85; p=0.03), being hyperglycemia the main associated criterion (*χ*2=11.7; p=0.001). In multivariable analysis, it was observed that the relationship was exclusive with the presence of type 2 diabetes mellitus (T2DM) OR: 3.22 (1.14-9.14); p=0.03.

**Conclusion:**

The relationship between ScH and MS in our population is dependent on the presence of hyperglycemia, specifically T2DM diagnosis, findings that vary from those previously reported in Latin American subjects.

## 1. Introduction

Metabolic Syndrome (MS) is defined as a “constellation” of cardiometabolic risk factors, which, jointly, increase the risk of suffering cardiovascular diseases and type 2 diabetes mellitus [1]. However, its presence has been related to a great number of alterations that go from cancer to sleep apnea, polycystic ovary syndrome, thyroid disruptions, amongst others [2, 3]. The high prevalence of MS is a worrying matter in various Latin American populations [4].

On the other hand, subclinical hypothyroidism (ScH) is defined by high thyroid stimulating hormone (TSH) levels with normal free thyroxine (T4) [5]. Although it has been associated with coronary and carotid arterial disease, the association between ScH and MS and its individual components is currently a controversial subject. There are inconsistent results as regards to the triggers of thyroid disruptions, aside from the autoimmune etiological process, the cut-off values for its diagnostic and the temporary association with individual effects of some metabolic alterations on its development [6].

Regarding this subject, various studies have demonstrated the existence of an association between increased TSH levels and the rise of Body Mass Index (BMI) and body fat percentage [7]. However, studies that evaluate the relation between these metabolic disturbances locally are limited; therefore, the purpose of this study was to analyze the association between ScH and MS components in the adult population from Maracaibo City, Venezuela.

## 2. Materials and Methods

### 2.1. Study Design and Population Selection

The Maracaibo City Metabolic Syndrome Prevalence Study (MMSPS) was a descriptive, cross-sectional study performed in Maracaibo City, Venezuela (May 2007-December 2009) with the purpose of determining the prevalence of MS and cardiovascular risk factors in adult population of this city; the study protocol has been previously reported [8]. The most important aspects are presented here; Maracaibo City was divided into parishes, which were sampled through a multistage random sampling process, defining conglomerates in two phases. In the first phase, conglomerates represented sectors of the 18 parishes, selecting 4 areas per parish by means of simple random sampling. In the second phase, the conglomerates were represented by the neighborhood of each chosen area, to which a random number was assigned. The study included finally 2,230 individuals of both genders, over 18 years old.

For this analysis, 425 patients whose thyroid profile had been biochemically evaluated were randomly selected in order to analyze ScH prevalence. For this selection, the random number tool of the SPSS software was used. The percentage of cases to evaluate was specified (approximately 19% of the total simple) and the selection of individuals was set equally between both genders. No other inclusion criterion was set.

**Figure 1:**
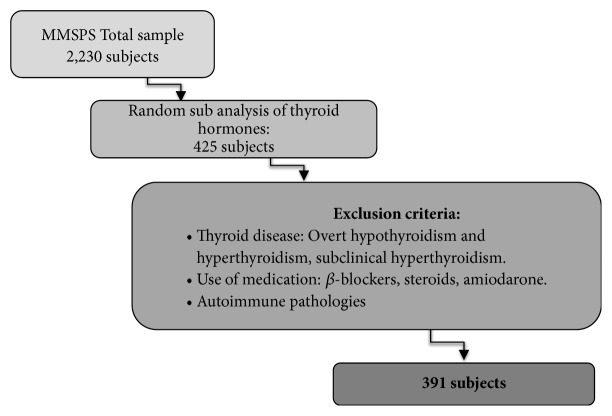
Algorithm for selection of the patients for subclinical hypothyroidism evaluation in adults from Maracaibo City, Venezuela.

The individuals that were receiving *β*-blockers (2.0%; n=9), steroids (2.0%; n=9), and amiodarone (0.2%; n=1) were excluded, as well as subjects with thyroid disease diagnoses: overt hypothyroidism (1.6%; n=7), subclinical hyperthyroidism (0.9%; n=4), overt hyperthyroidism (0.7%; n=3), and type 1 diabetes mellitus (0.2%, n=1). A final sample of 391 subjects was evaluated (Figure 1).

### 2.2. Ethical Considerations

The study was approved by the Bioethics Committee of the Endocrine and Metabolic Research Center, University of Zulia (approval number: BEC-006-0305). This ethical approval included all future studies that used the data from the MMSPS. All participants signed an informed written consent for participation in the study before being interviewed and physically examined by a trained team.

### 2.3. Evaluation of Individuals

All individuals were evaluated with a complete clinical history performed by trained personnel. Personal and family medical history was gathered, with emphasis on metabolic, endocrine, and cardiovascular diseases history. Sociodemographic characteristics such as age, gender, and socioeconomic status (classified as high class, middle-high class, middle class, working class, and extreme poverty according to the Graffar method, which uses monthly income and housing characteristics) were similarly studied [9].

### 2.4. Clinical Evaluation

An anthropometric evaluation was performed: weight was determined using a digital scale (Tanita, TBF-310 GS Body Composition Analyzer, Tokyo-Japan), while height was measured with a previously calibrated stadiometer on a flat surface. The Body Mass Index (BMI) was calculated using the formula [Weight (Kg) / (Height (meters))^2^], and individuals were categorized based on the World Health Organization (WHO) classification [10]. Waist circumference (WC) was measured with a plastic measuring tape graduated in centimeters and millimeters, according to the protocol proposed by the US National Health Institute (NHI) [11]. Blood pressure (BP) was measured through the auscultation method with subjects sitting down with their feet on the floor following a 15-minute rest, determined with a calibrated mercury sphygmomanometer. BP was determined 3 times, with 15 minutes in between each registration, on two different days.

### 2.5. Laboratory Analysis

After 8 hours of fasting, serum levels of glucose (catalog number REF-10123), total cholesterol (catalog number REF-10015), HDL-C (catalog number REF-10193), and triacylglycerides (TG) (catalog number REF-10163) were determined using enzymatic-colorimetric kits (Human Gesellschaft für Biochemica und Diagnostica mbH) and a specialized computer system. The intra- and interassay coefficients of variation (CV) were 3.3% for serum glucose, 3.3% for total cholesterol, 3.3% for HDL-C, and 5.5% for TG. Insulin was determined using a commercial kit (catalog number EIA-2935) based on the ELISA double-sandwich method (DRG Instruments GmbH, Germany), with a detection limit <1 mU/L and inter- and intra-assay CV was 10.0%.

Serum free T3 (catalog number REF-54015), free T4 (catalog number REF-54025), and TSH (catalog number REF-54030) were measured using a sandwich ELISA kit (Human Gesellschaft für Biochemica und Diagnostica mbH) as described by the manufacturer. Absorbance of standards and specimens was determined using ELISA microplate reader Anthos 2001 (Anthos Labtec Instruments GesmbH, Austria). The intensity of the color is directly proportional to the hormones concentration in the standards and samples. The intra-assay CV ranged from 2.0 to 5.4% for free T3 (ELISA tests analytic sensitivity: 0.05 ng/mL), 3.1-6.0% for free T4 (ELISA tests analytic sensitivity: 0.4 pg/dL), and 4.0-5.4% for TSH (ELISA tests analytic sensitivity: <0.10 mIU/L).

### 2.6. Definitions

The MS components according to IDF/AHA/NHLBI/WHF/IAS/IASO-2009 criteria [12] were

- elevated WC: ≥80 cm in females or ≥90 cm in males;
- high TG: ≥150 mg/dL;
- low HDL-C: <50 mg/dL in females or <40 mg/dL in males;
- high blood pressure: BP ≥130/85 mm/Hg or previously diagnosed hypertension or prescription of antihypertensive drugs; and
- hyperglycemia: fasting glycaemia ≥100 mg/dL, or personal history of T2DM or prescription of hypoglycemic drugs.

ScH diagnosis was obtained following the criteria used by the NHANES (National Health and Nutrition Examination Survey), which establishes normal T4 levels (0.9-1,9 pg/dL), together with high TSH levels (≥4.12mUI/L), with no personal records of known thyroid pathology [13]. The Homeostasis Model Assessment 2 (HOMA2-IR) was calculated using the HOMA-Calculator v2.2.2 software; the proposed cut-off point for our population was 2.0 to define IR [14]. Glycemic status was defined as T2DM, in patients with one of the following criteria: (a) previous diagnosis of T2DM; (b) no previous personal history of T2DM, but with fasting glucose ≥126 mg/dl; and impaired fasting glucose in patients with fasting glucose between 100 and 126 mg/dl [15].

### 2.7. Statistical Analysis

Qualitative variables were expressed in absolute and relative frequencies, evaluating the association between them using the *χ*^2^ test (Chi square). Quantitative variables were expressed as arithmetic means ± SD, after analysis of normality by Geary test. A multiple logistic regression model was performed for ScH (dependent variable), adjusted by age group, sex, socioeconomic status, and MS components. In successive models, these were adjusted according to specific glycemic status and presence of IR. All the data was analyzed with SPSS v.21 for Windows (IBM Chicago, IL). The results were considered statistically significant when p<0.05.

## 3. Results

### 3.1. General Characteristics of the Analyzed Sample

The prevalence of ScH in 391 evaluated subjects was 10.5% (n=41), with a higher frequency in women (women: 13.6% versus men: 7.7%; *χ*^2^=3.561, p=0.05), and 60 years or older individuals (26.1%, n=12). The general characteristics of the population are shown in Table 1.

### 3.2. Subclinical Hypothyroidism and Metabolic Syndrome

**Figure 2:**
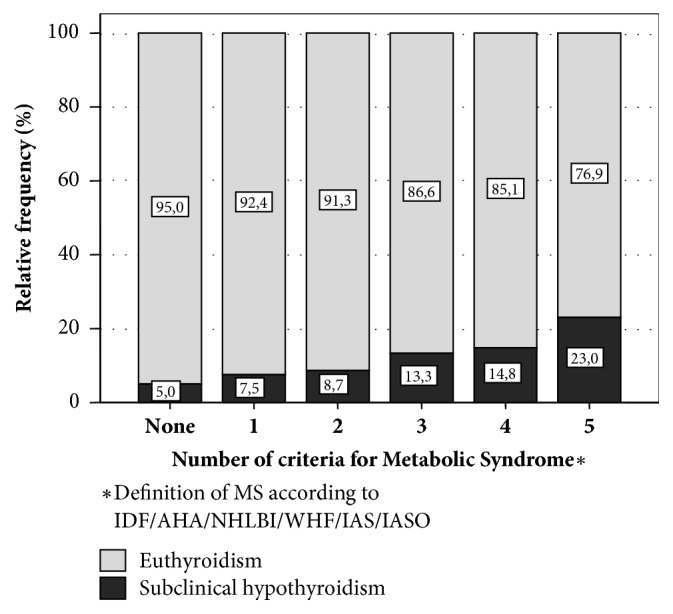
Subclinical hypothyroidism and number of criteria for Metabolic Syndrome in adults from Maracaibo City, Venezuela.

Among the individuals with ScH, 56.1% (n=23) had MS, a higher number than patients with euthyroidism: 38.3% (n=134); *χ*^2^=4.845, p=0.028, with an increase in percentage as the number of criteria rises (0 criteria: 5.0% versus 5 criteria: 23.0%; p=0.22) (Figure 2).

### 3.3. Subclinical Hypothyroidism and Metabolic Syndrome Components

When evaluating MS components, hyperglycemia was the most frequent metabolic alteration in subjects with ScH (without hyperglycemia: 8% versus 22.1% with hyperglycemia; *χ*2=11.7, p=0.001). The rest of the components can be seen on Table 2. As regards to thyroid hormones, only free T3 showed lower levels in subjects with hyperglycemia, high TG, and elevated WC (Table 3).

On the other hand, in the multivariate analysis, hyperglycemia was the only metabolic disorder related to ScH, showing that the presence of diabetes is associated with thyroid alteration (OR: 3.22; CI 95%: 1.14-9.14; p=0.03) (Table 4).

## 4. Discussion

ScH is a metabolic disorder that affects approximately 4-10% of the general US population [16], with its etiology residing mainly in autoimmune alterations. However, diverse reports have suggested that the presence of MS can lead to a higher risk for its appearance [6]. Our research team had previously determined a prevalence of 9.6% in the adult population of Maracaibo City, a region with enough iodine intake from regular diet. Similarly, this report did not show a positive association with ScH and MS when adjusting for a multivariate context of diverse cardiometabolic disorders [17].

Therefore, the purpose of this cross-sectional study was to determine the degree of association that exists between individual components of MS and ScH diagnosis in our sample, given the previous reported findings. In spite of a high prevalence of MS in patients with ScH, hyperglycemia alone was the metabolic factor that showed a positive correlation with thyroid disorders.

Our findings are similar to those reported by Garduño-Garcia et al. [18]. They determined in more than 3,000 Mexican subjects a lack of association between ScH and MS risk. However, there was a correlation between loss of thyroid function and higher cholesterol, glycaemia, insulin, and HOMA-IR levels. Similarly, Pesic et al. [19], in a case control study with 120 subjects, observed higher BMI, blood pressure, total cholesterol, serum TG, serum insulin, hypertension, and HDL-C levels in patients with ScH. However, when evaluating the prevalence of MS in both groups, there were no significant differences (normal thyroid hormone levels: 46.67% versus ScH: 33.3%; p>0.05). It is important to highlight that both reports excluded diabetic subjects.

Conversely, numerous studies differ with the previously mentioned results, showing a relation between individual MS component as well as with the presence of MS as a whole. This was observed in cross-sectional reports from Nepal [20], Nigeria [21], and Turkey [22]. It is interesting to observe that, in these analyses, glycemic alterations were not amongst the main related factors, an aspect that could be influenced by the stage in which the individual is classified (prediabetes, diabetes), therapeutic management, and the time of disease evolution. In this regard, interaction between diabetes and thyroid ailments has been widely described in studies both in vitro and in vivo, with IR as the main phenomenon responsible, implicated in both hyperthyroidism and hypothyroidism [23].

Regarding hypothyroidism, a frequent coexistence between both alterations, especially in subjects with subclinical hypothyroidism, has been observed [24]. Chaker et al. [25] have reported this recently, in a prospective analysis with more than 8,000 subjects that belonged to the Rotterdam Study, where a higher incidence of T2DM was observed in those subjects with low or normal to low thyroid function. Similarly, this association does not seem to be limited to early T2DM stages, but to also be related to the apparition of chronic complications such as diabetic nephropathy [26, 27].

It has been recognized that there is a narrow relationship between thyroid disease and T2DM, in which the following observable facts are underlying: homeostasis disorder of hepatic glycaemia and interaction with adipokines like adiponectin and leptin. These mechanisms explain the proclivity towards dysglyceamia, lipid partition disorder, and poor metabolic control in patients with both clinical presentations, together with a decrease of the effective concentrations of FT3 due to a reduction of the expression of deiodinase [28, 29]. In fact, it has been proposed that the increment of TSH in obesity is a paraphenomenon similar to insulin resistance, in which leptin is the responsible for rising levels of thyrotropin, being the resistance in TSH receptors a consequence of this process [30, 31].

Moreover, it is important to consider the effect of thyroid hormones over carbohydrate metabolism, decreasing the translocation of glucose transporters and therefore the usage of glycaemia inside the cell, which is expressed as lower sensitivity towards insulin, regulating glycogen synthesis, fatty acids, and the expression of protein kinases in skeletal muscles [32].

The previously described findings have led to two hypotheses that are still cause for controversy in the area of endocrinology: (1) Should the thyroid disorder screening strategy be modified in diabetic patients? (2) What benefits does thyroid hormone supplementation offer to this group of patients? In this context, only the Thyroid Disease Clinical Practice Guidelines, by the American Association of Clinical Endocrinologists, highlight thyroid palpation and TSH in diagnosis, especially if goiter or other autoimmune disease presents in association with T2DM. Regular screening for thyroid abnormalities in all diabetic patients will allow early treatment of subclinical thyroid dysfunction [33]. On the other hand, diverse studies have shown a positive effect in glycaemia regulation, including ScH subjects, with the usage of L-thyroxin [34].

There are various limitations in the present study. Initially, the cross-sectional design makes it impossible to establish causality in the analyzed variables. Therefore, a prospective analysis is necessary in order to confirm our findings and evaluate other aspects that have not been considered in the current subject: bigger sample size, time of diabetes diagnosis, other comorbidities, and nutrition information. Finally, it is necessary to incorporate the determination of leptin and reverse T3 in order to properly evaluate the hormonal axis.

We can conclude that, in the population from Maracaibo City, the association between MS and ScH depends on the presence of T2DM, which can act as a confusing factor in this analysis, with IR as the physiopathological event implicated in this association. Therefore, determining the presence of thyroid disorders, even in a subclinical way, is a strategy to consider in the diabetic patients of our region.

## Figures and Tables

**Table 1:** General characteristics according to the presence of subclinical hypothyroidism in adults from Maracaibo City, Venezuela.

	**Euthyroidism**		**Subclinical hypothyroidism**		**Total**		**χ** ^2^ ***(p)*** **∗**
	**n**	%	**n**	%	**n**	%	
**Sex**							**3,5 (0,059)**
Female	159	86,4	25	13,6	184	52,9	
Male	191	92,3	16	7,7	207	47,1	
**Age groups (years)**							**19,8 (<0,001)**
<40	183	95,3	9	4,7	192	49,1	
40-59	133	86,9	20	13,1	153	39,1	
≥60	34	73,9	12	26,1	46	11,8	
**Socioeconomic strata**							**7,55 (0,109)**
High class	5	62,5	3	37,5	8	2,0	
Middle-high class	56	88,9	7	11,1	63	16,2	
Middle class	157	91,8	14	8,2	171	43,7	
Working class	120	88,2	16	11,8	136	34,8	
Extreme poverty	12	92,3	1	7,7	13	3,3	
**T2DM§**							**12,21 (<0,001)**
Absent	332	94,9	33	80,5	365	93,4	
Present	18	5,1	8	19,5	26	6,6	
**Total**	**350**	**89,5**	**41**	**10,5**	**391**	**100,0**	

§ Type 2 diabetes mellitus personal history.*∗* Chi square test.

**Table 2:** Subclinical hypothyroidism and components of metabolic syndrome in adults from Maracaibo City, Venezuela.

	**Euthyroidism** **(n=350)**		**Subclinical Hypothyroidism** **(n=41)**		**Total** **(n=391)**		
	**n**	%	**n**	%	**n**	%	**χ** ^2^ ***(p)*** **∗**
**Hyperglycaemia**§							**11,7 (0,001)**
Absent	297	92,0	26	8,0	323	82,6	
Present	53	77,9	15	22,1	68	17,4	
**High triacylglycerides**†							**2,4 (0,116)**
Absent	262	91,0	26	9,0	288	73,7	
Present	88	85,4	15	14,6	103	26,3	
**High Blood Pressure**¥							**1,7 (0,191)**
Absent	208	91,2	20	8,8	228	58,3	
Present	142	87,1	21	12,9	163	41,7	
**Low HDL-C**¶							**0,2 (0,622)**
Absent	142	90,4	15	9,6	157	40,2	
Present	208	88,9	26	11,1	234	59,8	
**High Abdominal Circumference**Ψ							**0,2 (0,600)**
Absent	90	90,9	9	9,1	99	25,3	
Present	260	89,0	32	11,0	292	74,7	

§ Fasting glycaemia ≥100mg/dL, or personal history of DM2 or prescription of hypoglycemic drugs.† TG ≥150mg/dL. ¥ Blood pressure ≥130/85mm/Hg or previously diagnosed hypertension or prescription of antihypertensive drugs.¶ HDL-C <50mg/dL in females or <40mg/dL in males.Ψ WC ≥80 cm in females or ≥90 cm in males.*∗* Chi square test. HDL-C: high density lipoprotein.

**Table 3:** Thyroid hormones and metabolic syndrome components in adults from Maracaibo City, Venezuela.

	**TSH (mUI/L)**			**Free T**_**3 **_ **(ng/mL)**			**Free T**_**4 **_ **(pg/gL)**		
	**Mean**	**SD**	***p*** **∗**	**Mean**	**SD**	***p*** **∗**	**Mean**	**SD**	***p*** **∗**
**Hyperglycaemia**§			0,638			**0,020**			0,856
Absent	8,05	7,00		2,94	0,73		1,22	0,20	
Present	7,15	3,06		2,41	0,57		1,21	0,23	
**High triacylglycerides**†			0,728			**0,011**			0,903
Absent	7,48	5,81		2,96	0,73		1,21	0,20	
Present	8,15	6,07		2,38	0,55		1,22	0,23	
**High Blood Pressure**¥			0,604			0,133			0,456
Absent	8,22	6,81		2,92	0,70		1,24	0,21	
Present	7,25	4,87		2,58	0,71		1,19	0,21	
**Low HDL-C**¶			0,247			0,397			0,523
Absent	6,45	4,18		2,87	0,79		1,19	0,17	
Present	8,45	6,58		2,67	0,68		1,23	0,23	
**High Abdominal Circumference**Ψ			0,352			**0,010**			0,766
Absent	9,34	8,30		3,28	0,71		1,23	0,15	
Present	7,27	5,03		2,60	0,66		1,21	0,22	

§ Fasting glycaemia ≥100mg/dL, or personal history of DM2 or prescription of hypoglycemic drugs.† TG ≥150mg/dL. ¥ Blood pressure ≥130/85mm/Hg or previously diagnosed hypertension or prescription of antihypertensive drugs.¶ HDL-C <50mg/dL in females or <40mg/dL in males.Ψ WC ≥80 cm in females or ≥90 cm in males.*∗* Student's t-test. HDL-C: high density lipoprotein.

**Table 4:** Metabolic risk factors for subclinical hypothyroidism in adults from Maracaibo City, Venezuela.

	**Model 1** **∗**				**Model 2** **∗** **∗**	
	**Odds Ratio** **(IC 95**%^**a**^**)**	**p** ^**b**^	**Adjusted Odds Ratio ** **(IC 95**%^**a**^**)**	**p** ^**b**^	**Adjusted Odds Ratio ** **(IC 95**%^**a**^**)**	**p** ^**b**^
**High Abdominal Circumference**†						
Absent	1,00	-	1,00	-	1,00	-
Present	1,23 (0,57 - 2,68)	0,60	0,67 (0,28 - 1,61)	0,37	0,71 (0,29 - 1,75)	0,46
**High Blood Pressure**†						
Absent	1,00	-	1,00	-	1,00	-
Present	1,64 (1,35 - 1,99)	<0,01	0,91 (0,42 - 1,94)	0,80	0,87 (0,41 - 1,87)	0,73
**Low HDL-C**†						
Absent	1,00	-	1,00	-	1,00	-
Present	1,18 (0,61 - 2,31)	0,62	1,01 (0,48 - 2,10)	0,99	0,99 (0,47 - 2,09)	0,99
**High triacylglycerides**†						
Absent	1,00	-	1,00	-	1,00	-
Present	1,72 (0,87 - 3,39)	0,12	1,32 (0,62 - 2,84)	0,47	1,33 (0,61 - 2,88)	0,47
**Hyperglycaemia**†						
Absent	1,00	-	1,00	-	-	-
Present	3,23 (1,61 - 6,51)	< 0,01	2,20 (1,00 - 4,89)	0,05	-	-
**Glycemic status**						
Normoglycemic	1,00	-	-	-	1,00	-
Impaired Fasting Glucose	2,29 (0,87 - 5,99)	0,09	-	-	**1,31 (0,39 - 4,42)**	**0,67**
Type 2 Diabetes Mellitus	4,47 (1,88 - 10,66)	< 0,01	-	-	**3,22 (1,14 - 9,14)**	**0,03**

† IDF/AHA/NHLBI/WHF/IASO-2009 criteria with a confidence interval of 95%, b level of significance.**∗ Model 1**: sex, age groups, socioeconomic status, and metabolic syndrome components. **∗∗ Model 2**: similar adjust with specific glycemic status and insulin resistance.

## Data Availability

The database will be sent by the corresponding author (Dr. Valmore Bermúdez: valmore@gmail.com) to the reviewers, editors, and researchers who wish to reproduce the results.

## References

[1] Nolan P. B., Carrick-Ranson G., Stinear J. W., Reading S. A., Dalleck L. C. (2017). Prevalence of metabolic syndrome and metabolic syndrome components in young adults: A pooled analysis. *Preventive Medicine Reports*.

[2] Delitala A. P., Fanciulli G., Pes G. M., Maioli M., Delitala G. (2017). Thyroid hormones, metabolic syndrome and its components. *Endocrine, Metabolic & Immune Disorders—Drug Targets*.

[3] Esposito K., Chiodini P., Colao A., Lenzi A., Giugliano D. (2012). Metabolic syndrome and risk of cancer: a systematic review and meta-analysis. *Diabetes Care*.

[4] Cuevas A., Álvarez V., Carrasco F. (2011). Epidemic of metabolic syndrome in Latin America. *Current Opinion in Endocrinology, Diabetes and Obesity*.

[5] Surks M. I., Ortiz E., Daniels G. H. (2004). Subclinical thyroid disease: scientific review and guidelines for diagnosis and management. *Journal of the American Medical Association*.

[6] Chang C., Yeh Y., Caffrey J. L., Shih S., Chuang L., Tu Y. (2017). Metabolic syndrome is associated with an increased incidence of subclinical hypothyroidism – A Cohort Study. *Scientific Reports*.

[7] Fox C. S., Pencina M. J., D'Agostino R. B. (2008). Relations of thyroid function to body weight: Cross-sectional and longitudinal observations in a community-based sample. *JAMA Internal Medicine*.

[8] Bermúdez V., Marcano R. P., Cano C. (2010). The maracaibo city metabolic syndrome prevalence study: design and scope. *American Journal of Therapeutics*.

[9] Méndez Castellano H., de Méndez M. C. (1986). Estratificación social y biología humana: método Graffar modificado. *Arch Ven Pueric Pediatr*.

[10] World Health Organization (2000). Obesity: preventing and managing the global epidemic. Report of a WHO Consultation on Obesity. *Geneva: The Organization (WHO Technical Report Series, No. 894)*.

[11] Centers for Disease Control and Prevention NHANES - NHANES III - Reports and Reference Manuals. http://www.cdc.gov/nchs/nhanes/nh3rrm.htm.

[12] Alberti K. G., Eckel R. H., Grundy S. M. (2009). Harmonizing the metabolic syndrome: a joint interim statement of the international diabetes federation task force on epidemiology and prevention; National heart, lung, and blood institute; American heart association; World heart federation; International atherosclerosis society; and international association for the study of obesity. *Circulation*.

[13] Hollowell J. G., Staehling N. W., Flanders W. D. (2002). Serum TSH, T4, and Thyroid Antibodies in the United States Population (1988 to 1994): National Health and Nutrition Examination Survey (NHANES III). *The Journal of Clinical Endocrinology & Metabolism*.

[14] Bermudez V., Rojas J., Martinez M. S., etal. (2014). Epidemiologic Behavior and Estimation of an Optimal Cut-Off Point for Homeostasis Model Assessment-2 Insulin Resistance: A Report from a Venezuelan Population. *International Scholarly Research Notices*.

[15] American Diabetes Association (2017). Standards of medical care in diabetes—2017: summary of revisions. *Diabetes Care*.

[16] Biondi B., Cooper D. S. (2008). The clinical significance of subclinical thyroid dysfunction. *Endocrine Reviews*.

[17] Bermúdez V., Cabrera M., Chavez C. (2013). Comportamiento epidemiológico del hipotiroidismo subclínico y su asociación con factores de riesgo cardiometabólicos en individuos adultos del municipio Maracaibo, Venezuela. *Revista Latinoamericana de Hipertensión*.

[18] Garduño-Garcia J. D. J., Alvirde-Garcia U., López-Carrasco G. (2010). TSH and free thyroxine concentrations are associated with differing metabolic markers in euthyroid subjects. *European Journal of Endocrinology*.

[19] Pesic M. M., Radojkovic D., Antic S., Kocic R., Stankovic-Djordjevic D. (2015). Subclinical hypothyroidism: Association with cardiovascular risk factors and components of metabolic syndrome. *Biotechnology & Biotechnological Equipment*.

[20] Khatiwada S., Sah S. K., KC R., Baral N., Lamsal M. (2016). Thyroid dysfunction in metabolic syndrome patients and its relationship with components of metabolic syndrome. *Clinical Diabetes and Endocrinology*.

[21] Ogbera A., Dada O., Kuku S. (2012). The metabolic syndrome in thyroid disease: A report from Nigeria. *Indian Journal of Endocrinology and Metabolism*.

[22] Uzunlulu M., Yorulmaz E., Oguz A. (2007). Prevalence of subclinical hypothyroidism in patients with metabolic syndrome. *Endocrine Journal*.

[23] Wang C. (2013). The Relationship between Type 2 Diabetes Mellitus and Related Thyroid Diseases. *Journal of Diabetes Research*.

[24] Vázquez M., Rojas J., Bermudez V. (2013). Comportamiento epidemiológico del hipotiroidismo en pacientes con diabetes mellitus tipo 2 en la ciudad de Loja – Ecuador. *Revista Latinoamericana de Hipertension*.

[25] Chaker L., Ligthart S., Korevaar T. I. M. (2016). Thyroid function and risk of type 2 diabetes: A population-based prospective cohort study. *BMC Medicine*.

[26] Furukawa S., Yamamoto S., Todo Y. (2014). Association between subclinical hypothyroidism and diabetic nephropathy in patients with type 2 diabetes mellitus. *Endocrine Journal*.

[27] Han C., He X., Xia X. (2015). Subclinical hypothyroidism and type 2 diabetes: a systematic review and meta-analysis. *PLoS ONE*.

[28] Hage M., Zantout M. S., Azar S. T. (2011). Thyroid disorders and diabetes mellitus. *Journal of Thyroid Research*.

[29] Cabanelas A., Lisboa P. C., Moura E. G., Pazos Moura C. C. (2006). Leptin acute modulation of the 5′-deiodinase activities in hypothalamus, pituitary and brown adipose tissue of fed rats. *Hormone and Metabolic Research*.

[30] Topsakal S., Yerlikaya E., Akin F., Kaptanoglu B., Erürker T. (2012). Relation with HOMA-IR and thyroid hormones in obese Turkish women with metabolic syndrome. *Eating and Weight Disorders*.

[31] Han C., Li C., Mao J., etal. (2015). High Body Mass Index Is an Indicator of Maternal Hypothyroidism, Hypothyroxinemia, and Thyroid-Peroxidase Antibody Positivity during Early Pregnancy. *BioMed Research International*.

[32] Maratou E., Hadjidakis D. J., Kollias A. (2009). Studies of insulin resistance in patients with clinical and sub clinical hypothyroidism. *European Journal of Endocrinology*.

[33] Baskin H. J., Cobin R. H., Duick D. S. (2002). American association of clinical endocrinologists medical guidelines for clinical practice for the evaluation and treatment of hyperthyroidism and hypothyroidism. *Endocrine Practice*.

[34] Bilic-Komarica E., Beciragic A., Junuzovic D. (2012). Effects of treatment with L-thyroxin on glucose regulation in patients with subclinical hypothyroidism. *Medical Archives*.

